# Diurnal transcriptional variation is reduced in a nitrogen-fixing diatom endosymbiont

**DOI:** 10.1093/ismejo/wrae064

**Published:** 2024-04-18

**Authors:** Heidi Abresch, Tisza Bell, Scott R Miller

**Affiliations:** Division of Biological Sciences, The University of Montana, Missoula, MT 59812, United States; Division of Biological Sciences, The University of Montana, Missoula, MT 59812, United States; Division of Biological Sciences, The University of Montana, Missoula, MT 59812, United States

**Keywords:** endosymbiosis, nitrogen fixation, Cyanobacteria, diatoms, protist-prokaryote symbiosis, diatom-diazotroph associations

## Abstract

Many organisms have formed symbiotic relationships with nitrogen (N)-fixing bacteria to overcome N limitation. Diatoms in the family *Rhopalodiaceae* host unicellular, N-fixing cyanobacterial endosymbionts called spheroid bodies (SBs). Although this relationship is relatively young, SBs share many key features with older endosymbionts, including coordinated cell division and genome reduction. Unlike free-living relatives that fix N exclusively at night, SBs fix N largely during the day; however, how SB metabolism is regulated and coordinated with the host is not yet understood. We compared four SB genomes, including those from two new host species (*Rhopalodia gibba* and *Epithemia adnata*), to build a genome-wide phylogeny which provides a better understanding of SB evolutionary origins. Contrary to models of endosymbiotic genome reduction, the SB chromosome is unusually stable for an endosymbiont genome, likely due to the early loss of all mobile elements. Transcriptomic data for the *R. gibba* SB and host organelles addressed whether and how the allocation of transcriptional resources depends on light and nitrogen availability. Although allocation to the SB was high under all conditions, relative expression of chloroplast photosynthesis genes increased in the absence of nitrate, but this pattern was suppressed by nitrate addition. SB expression of catabolism genes was generally greater during daytime rather than at night, although the magnitude of diurnal changes in expression was modest compared to free-living *Cyanobacteria*. We conclude that SB daytime catabolism likely supports N-fixation by linking the process to host photosynthetic carbon fixation.

## Introduction

Diatoms are unicellular stramenopile algae with enormous impact on both marine and freshwater ecosystems that are responsible for at least 20% of global primary production [1]. In many environments, obtaining sufficient nitrogen (N) is a challenge, and diatoms use different mechanisms to do so. For example, some taxa vertically migrate through the water column to obtain nitrate at depth [2]. Moreover, multiple diatom genera have independently established close relationships with nitrogen (N)-fixing *Cyanobacteria*, broadly called diatom-diazotroph associations. Examples include endosymbioses with heterocystous *Cyanobacteria* (*Hemiaulus—Richelia intracellularis*, *Rhizosolenia*—*Calothrix rhizosolenia*) and between the centric diatom *Climacodium frauenfeldianum* and a unicellular cyanobacterium related to *Crocosphaera watsonii* [3].

Diatom-diazotroph associations have long been recognized as important for global carbon and nitrogen cycles, particularly in nutrient-poor marine environments [3]. These associations contribute a significant input of N into otherwise N-poor systems, such as the North Pacific gyre and the tropical North Atlantic [3–5]. These organisms make up a significant portion of the annual summer peaks in carbon sinking in the North Pacific Ocean [6]. Large river outputs, such as the Amazon River, also stimulate growth of these organisms, providing a source of new N and increasing carbon sequestration [7]. However, this research has primarily been focused on heterocystous symbionts in marine environments, particularly *R. intracellularis* and *C. rhizosolenia* [7–9]*.* Additionally, although these relationships are old (66–100 million years), they are typically not required for the host’s survival [4, 9, 10].

One of the closest diatom-diazotroph associations is in the family *Rhopalodiaceae—*primarily the genera *Epithemia* and *Rhopalodia*—which host unicellular cyanobacterial endosymbionts called spheroid bodies (SBs). These partners are already tightly associated, even though it is apparently more recent (at least 34 million years old based on the fossil record [11]). As a consequence of this relationship, *Rhopalodia* and *Epithemia* species are able to thrive in freshwater environments with a low nitrogen to phosphorus ratio around the world, and they can have major impacts on local food web structure [12–14]. Recently, members of *Rhopalodiaceae* containing SBs have also been shown to be widely distributed in marine environments [15]. Given their widespread distribution across freshwater and marine environments, the *Rhopalodiaceae*-SB association may be a more important contributor to global N-fixation than previously recognized.

Despite the comparatively recent origin of the *Rhopalodiaceae-*SB association, SBs already exhibit traits thought to be essential for long-lasting endosymbiotic relationships [16, 17], such as those between bacteria and sap-feeding insects [18]. SBs are uniparentally inherited during host sexual reproduction [19]. SB genome size has been reduced by nearly half compared to its closest free-living relative [16]. SBs have also lost key genes in metabolic pathways necessary for an independent phototrophic lifestyle, including nearly all those required for photosynthesis [20]. SBs have retained N-fixation genes, but unlike their free-living relatives, SBs fix N during the day alongside host photosynthesis and into the night [15, 16]. As nitrogenase is extremely sensitive to oxygen, daytime N-fixation is unexpected for single-celled *Cyanobacteria*, whereas heterocystous N-fixing *Cyanobacteria* (such as *Richelia* and *Calothrix* endosymbionts) enable daytime N-fixation through the spatial separation of N-fixation and oxygenic photosynthesis. The reasons for the shift in SB N-fixation are unknown, as are the mechanisms that make concurrent host photosynthesis and SB N-fixation possible.

Although endosymbioses are widespread and common in unicellular hosts, most investigations of endosymbiont evolution to date have focused on associations with animals or plants [21]. From those studies, there are many well-understood examples of older, highly integrated symbionts with extremely reduced genomes [18]. By contrast, there are few well-described examples of symbionts early in the host-integration process, before cellular processes are highly coordinated and endosymbiont genomes are highly reduced (mostly from insect hosts [22]). The *Rhopalodiaceae*-SB system therefore provides a remarkable opportunity to understand the early stages of endosymbiosis in a unicellular host, with respect to both the genome reduction process and how gene expression changes during the process of host integration.

In this study, we sequenced SB genomes from two new host species and used these, together with two publicly available SB genomes, to investigate how the SB has evolved over time. By comparing SB genomes to those of closely related, free-living *Cyanobacteria*, we were able to better determine where SBs are on the trajectory of endosymbiotic genome reduction and to predict their core metabolic capacity. Next, we obtained transcriptomic data for an SB and host organelles to address how transcriptional resources allocated among the SB and organelles, identify links to available N in the environment, and identify changes in SB expression of canonically diurnally regulated genes in concert with SB N-fixation.

## Materials and methods

### Cell isolation and culturing

*Rhopalodia gibba* and *Epithemia adnata* were isolated from *Cladophora glomerata* streamer mat samples collected from the Clark Fork River, Montana, USA, near Bonita Station Road in August 2017 (*R. gibba*) and August 2019 (*E. adnata*). Environmental samples were enriched in CSi-N liquid media, isolated by further dilutions and grown on 1% agar CSi-N plates. CSi-N is a modified CSi medium [17], lacking combined N (nitrate) and supplemented with 10 mM HEPES pH 8.0. Once isolated, cells were grown in 250 ml flasks with 125 ml media, maintained at 20°C on a 12:12-h light:dark cycle, and transferred every 6 weeks. Cultures are unialgal and uni-eukaryotic, but not axenic, as we have not been able to remove all bacterial contaminants.

### DNA extraction and sequencing

Cells were pelleted and resuspended in 900 μL TE and 100 μL of 1% Triton-X (in TE) to wash away the majority of exogenous bacteria before DNA extraction. DNA was extracted using the Qiagen DNeasy PowerBiofilm kit according to the manufacturer instructions. Libraries for short reads were prepared using a Nextera Flex DNA Library kit. Paired end short-read sequencing was performed with MiSeq500 v2 (Illumina). For *R. gibba*, high molecular weight DNA was also extracted using the protocol described elsewhere [23], prepped using a MinION Ligation Sequencing Kit, and sequenced using a FLO-MIN106 flow cell (Oxford Nanopore). All library preparations and sequencing were performed at the University of Montana Genomics Core.

### Genome assembly and annotations

We first assembled metagenomes in SPAdes version 3.12.0 [24] with auto-selected kmers of 21, 33, 55, 77, 99, and 127. For the *E. adnata* 19Bon2 assembly, we only used short-read data from a single sequencing run, resulting in 15 451 526 paired reads (SRR25945676). For the *R. gibba* 17Bon1 assembly, we used short-read data from three separate short-read sequencing runs for a total of 16 489 549 paired reads (SRR26035200, SRR26035201) and long-read sequences from a single long-read run (SRR26035202). SB, mitochondrion, and chloroplast scaffolds were then identified by BLAST sequence similarity to previously published SB and diatom sequences, further manually refined, and binned individually for downstream analyses. The *E. adnata* SB chromosome was assembled into a single closed scaffold with three internal gaps spanning complex tandem repeats that were unresolved by SPAdes. The *R. gibba* SB chromosome and both SB plasmids were assembled into single closed circles. Assemblies for both genomes were confirmed for correct circularization and consistent coverage by aligning raw reads to the assemblies using Bowtie [25]. We annotated SB genomes with the NCBI Prokaryotic Genome Annotation Pipeline (PGAP) [26]. Mitochondrion and chloroplast genomes were annotated using RAST (https://rast.nmpdr.org/). Mitochondria annotation for *R. gibba* 17Bon1 was further refined manually for downstream analyses. Additionally, the more recent PGAP annotations of *E. turgida *SB and *Rhopalodia gibberula SB* were used for all analyses.

### Phylogenetic reconstructions

We used OrthoFinder [27] to identify, align, and concatenate 885 single copy ortholog protein sequences (433 454 amino acid sites) shared by all taxa. A maximum likelihood phylogeny with 1000 ultrafast bootstrap replicates was constructed in IQTree version 1.7-beta9 using the cpREV+F + I + G4 model of evolution selected by ModelFinder [28–30]. Tree branches were tested by SH-like aLRT with 1000 replicates. Phylogeny figures were created in iTOL [31].

### Genome comparisons

SB genome synteny was assessed with Mauve version 20150226 build 10 using progressiveMauve [32]. Pan and core genomes were determined with Roary version 3.12.0 using a minimum BLASTp identity of 75% [33]. The minimum BLASTp identity for establishing the core genome was determined by making a histogram of the percent identity of best BLASTp hit comparisons between RulaSB and EadnSB to determine where percent identity was drastically reduced between the pair. The core genome was quality checked by manually inspecting genes that were (1) between 75%–80% percent similar, (2) shorter than 200 bp, or (3) where the shortest gene length was <80% of maximum gene length. For the core genome, we used eggNOG to assign Clusters of Orthologous Genes categories and KO (Kyoto Encyclopedia of Genes and Genomes [KEGG] Orthology) IDs to annotated SB genes with default settings [34]. A total of 976 of the 1515 core genes were assigned at least one KO ID. Complete pathways were identified using the KEGG Mapper [35].

### Transcriptome experiment

Eight 1 L flasks of *R. gibba* 17Bon1 were grown in 400 ml of CSi-N media in 12-h light:dark cycles. Seventy-two hours after inoculation, 10 mg/ml KNO_3_ was added to four of the flasks. Twenty-four hours after the addition of nitrate, 30 ml of culture was taken from each flask for the first mid-day RNA extraction and again 12 h later for mid-dark RNA. Two days later, 30 ml of culture was again extracted at mid-light and mid-dark for RNA extraction. At each time point, cells were pelleted and immediately frozen in liquid nitrogen and stored at −80°C until extraction. RNA was extracted using the Omega EZNA Plant RNA extraction kit, and libraries were prepped using Zymo-Seq RiboFree Total RNA Library Kit. Extracted reads were then sequenced using NovaSeq 6000 S4 (Illumina).

Raw reads were trimmed with trimmomatic v0.36 [36] to remove any low-quality reads using default settings. Paired reads were then combined when possible using flash version 1.2.11 [37]. Sortmerna version 4.3.4 [38] was used to remove any rRNA reads. Remaining reads were then aligned to the *R. gibba* 17Bon1 SB, mitochondrial, and chloroplast genomes using bowtie2 version 2.3.4.3 [25]. Reads were counted for protein coding sequence (CDS), transfer RNA (tRNA), and pseudogene features using htseq-count version 2.0.2 [39]. Final read counts were then analyzed to identify differentially expressed genes using DESeq2 [40] in R version 4.2.1. A summary of models used in data comparisons made in DESeq2 can be found in Supplementary Table 3.

## Results and discussion

### SB genome characteristics and phylogeny

**Figure 1:**
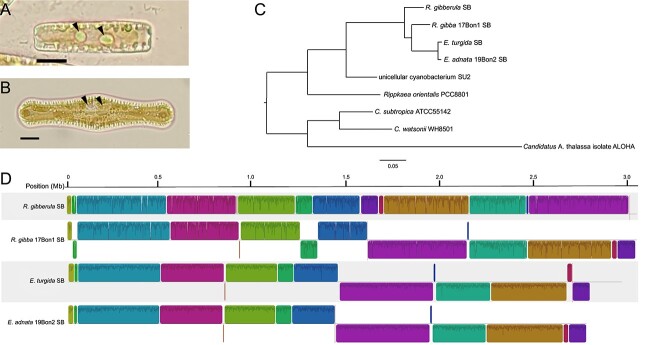
Light microscopy images of *R. gibba* 17Bon1 (A) and *E. adnata* 19Bon2 (B) with arrows indicating SBs; scale bars represent 10 μm; (C) maximum likelihood amino acid phylogeny reconstructed from a concatenated alignment of 885 single copy orthologs shared by all taxa using the cpREV+F+I+G4 model of evolution and was manually rooted based on the relationships in Shih *et al.* [59]; all nodes have 100% bootstrap support; scale bar is 0.05 expected amino acid substitutions per site; (D) synteny of SB chromosomes; homologous blocks of aligned sequence share the same color, and traces within a block indicate sequence similarity; blocks below the center line for each genome represent inversions compared to the reference strain (*R. gibberula* SB).

We obtained genome sequence data using both Illumina and Nanopore platforms for the SBs of *R. gibba* 17Bon1 (Fig. 1A) and *E. adnata* 19Bon2 (Fig. 1B), two recently isolated laboratory strains from the Clark Fork River, MT*.* Both the *R. gibba* SB (RgibSB) and the *E. adnata* SB (EadnSB) assembled into closed circular chromosomes (Supplementary Fig. 1) that are similar in size and guanine-cytosine (GC) content (Table 1) to two previously published SB genomes from *E. turgida* (EturSB; [20]) and *R. gibberula* (RulaSB; [41]). We also assembled a small circular plasmid in each SB. Previous studies [20, 41] described similarly sized contig sequences in two SB assemblies but did not examine them further. All four plasmids encode the same five genes, including *feoAB* genes (for the uptake of ferrous iron) and an annotated aquaporin Z gene (*aqpZ*). All SBs also have similar numbers of predicted genes and pseudogenes and are identical in rRNA operon copy number and tRNA content. Summary statistics for the four SB genomes and that of a close, free-living relative (*Rippkaea orientalis* PCC 8801, previously *Cyanothece* sp. PCC 8801 [42]) are given in Table 1. In addition to the SB genomes, we were also able to assemble and annotate nearly complete chloroplast and mitochondrion genomes for both host strains (Supplementary Table 1).

**Table 1:** Summary of genome content for SBs and close, free-living relative *Rippkaea orientalis* PCC 8801. Asterisk indicates a scaffold with unresolved repeats.

***Genome Information***	***Scaffolds***	***Total genome size (bp)***	***Chromosome Size (bp)***	***GC (%)***	***rRNA gene cluster***	***tRNAs***	***ncRNAs***	***Predicted protein coding genes***	***Coding density (genes/kilobase)***	***Predicted pseudogenes***	***Pseudogenes (%)***	***SB plasmid size (bp)***	***Plasmid GC (%)***	***Plasmid predicted protein coding genes***	***Accession number***
***E. adnata* 19Bon2 SB**	1 chromosome, 1 plasmid	27 88 257	2782614*	33.8	2	39	4	1671	0.601	126	7.0	5643	25.8	5	CP076462 (chromosome) CP076463 (plasmid)
***R. gibba* 17Bon1 SB**	1 chromosome, 1 plasmid	30 64 602	30 58 178	33.9	2	39	4	1776	0.581	148	7.7	6424	26.4	5	CP067995 (chromosome) CP067996 (plasmid)
***R. gibberula* SB**	1 chromosome, 1 plasmid	30 26 386	30 20 309	33.9	2	39	4	1848	0.612	99	5.1	6077	27.2	5	NZ_AP018341.1
***E. turgida* SB**	1 chromosome, 1 plasmid	28 00025	27 94 318	33.4	2	39	4	1664	0.595	137	7.6	5707	23.9	5	NZ_AP012549.1
***R. orientalis* PCC 8801**	1 chromosome, 3 plasmids	47 87 694	46 79 413	39.8	2	45	4	4397	1.064	86	2.0		-		GCF_000021805.1

We used maximum likelihood to construct a genome-wide amino acid phylogeny of the SBs, along with three closely related free-living *Cyanobacteria* and *Candidatus* Atelocyanobacterium thalassa isolate ALOHA (UCYN-A), the single-celled, N-fixing symbiont of a unicellular marine haptophyte [43]. As expected, the SBs formed a clade (Fig. 1C). The closest known relatives of the SBs are the unicellular, N-fixing strain SU2, isolated from coastal Zanzibar, Tanzania (GenBank accession number: GCA_002110465.1), and *R. orientalis* PCC 8801 (Fig. 1C). For further comparative analyses, we focused on *R. orientalis* PCC 8801, because it is a well-studied strain with a complete and closed genome [44]. This phylogeny differs from previously published SB trees reconstructed from limited data (i.e. single genes) [15–17], which placed SBs as sister to either *Crocosphaera subtropica* ATCC 51142 or *Ca*. Atelocyanobacterium thalassa isolate ALOHA (UCYN-A). Consequently, our phylogeny provides an improved hypothesis of SB origin and evolutionary history.

### SB genome reduction

The genomes of endosymbionts are predicted to follow a common evolutionary trajectory as they transition from a free-living to an obligate intracellular lifestyle [18]. Due to the stability of the host environment, selection is relaxed on many genes (such as those for sensing and responding to environmental changes), which are subsequently lost through deletional bias [45]. Single-celled photosynthetic hosts may have a more dynamic intracellular environment due to daily cycles in photosynthesis. However, endosymbionts are still buffered from changes in the external environment such as nutrient availability. Selection to maintain genes is also weaker due to low endosymbiont population sizes, which increases the influence of genetic drift on genome evolution [18]. Consequently, endosymbiont genomes get smaller over time. Current models predict that recently host-restricted endosymbiont genomes rapidly shrink, have significantly increased mobile elements and pseudogenes, and are subject to frequent chromosome rearrangements [18]. By contrast, long-term obligate endosymbionts have very few pseudogenes, no mobile elements, and typically more stable chromosomes (but this is not a universal trait, see Campbell *et al.*[46]). It is hypothesized that early proliferation of insertion sequences (ISs) provides substrates for large-scale deletions through recombination, driving rapid genome reduction early in the endosymbiosis [18]. From this, we would also predict that endosymbiont genomes would be highly reduced prior to IS element loss. Analysis of SB genomes can further our understanding of the early dynamics and drivers of endosymbiont genome reduction.

We expect SB genomes to closely resemble recently host-restricted endosymbionts based on their comparatively recent origin and large genomes. In agreement with this model, SB genomes have a very low coding density compared with free-living relatives (Table 1). This can partly be attributed to enriched pseudogene content, which is more than twice that of *R. orientalis* PCC 8801 (Table 1). Non-coding regions likely contain highly degraded gene remnants that are not easily identifiable. Elevated pseudogene content and idiosyncratic differences in retained genes among SB genomes (see below) suggest that SBs are still in the active genome reduction phase.

**Figure 2:**
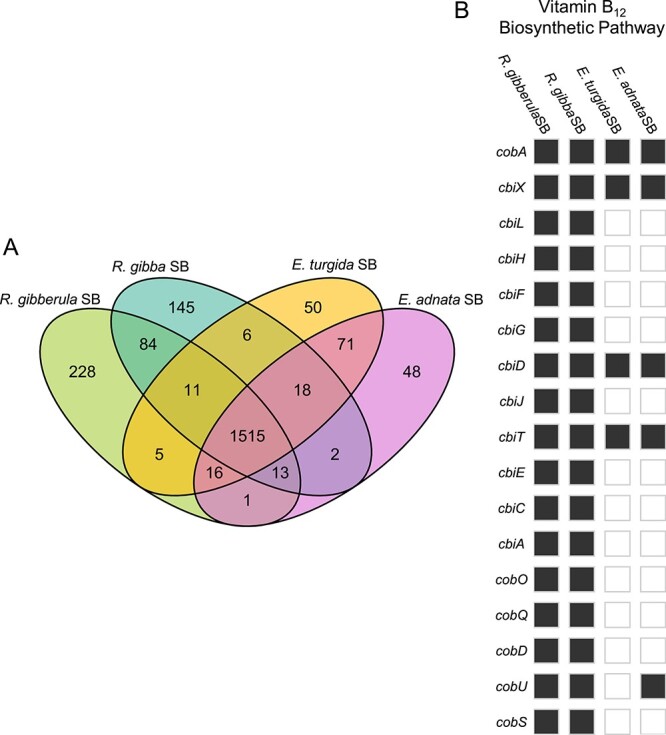
(A) Shared and unique gene content of SB genomes; (B) presence and absence of genes in the B_12_ biosynthetic pathway in SBs; grey and white indicate presence and absence, respectively.

In contrast to what is expected of young endosymbionts, SB chromosomes appear highly stable and have no mobile elements. A multiple genome-wide alignment shows SBs have a high degree of synteny and minimal genomic rearrangements (Fig. 1D). We propose that this unexpected stability is due to the early loss of all IS elements in SBs. Both the *R. orientalis* PCC 8801 genome and the unicellular cyanobacterium SU2 assembly contain at least 75 and 61 transposases respectively, whereas only fragments of transposases are present in SB genomes (Supplementary Table 2). The largest identifiable element in SBs is a truncated copy (~100 AA) of an ancestral IS3 family element that is retained as a single copy in all SB genomes and does not appear to be active. This indicates that the genome of the SB ancestor contained IS elements that were lost early during SB genome reduction. Recombination and repair genes are also mostly present in SB genomes (Supplementary Fig. 2); this is uncommon in the genomes of host-beneficial endosymbionts [21] and likely further promotes chromosome stability. Consequently, we predict that SB genomes will continue to shrink at a much slower pace than would otherwise be expected. The unique gene content among SB genomes suggests that there is still idiosyncratic, stochastic gene loss occurring in each SB genome (Fig. 2A; discussed below).

### SB metabolic capacity is mostly conserved across lineages

We analyzed SB gene content to better understand which core functions SBs have retained. Previous studies compared RulaSB and EturSB and found few differences between core metabolic pathways [41]. Our addition of two new SB genomes to this analysis strengthens this viewpoint. All SBs share a core of 1515 genes (including the five genes from the conserved plasmid), which is 80%–90% of the gene content of any individual SB (Fig. 2A). All functionally annotated genes present in SBs are also present in at least one of *R. orientalis* PCC 8801 or SU2, and usually both, with a single exception. This agrees with the expectation that endosymbionts do not gain new genes through horizontal gene transfer [18, 47]. The exception was a cyclophilin, *cypA* (annotated as peptidylprolyl isomerase), which is found in all SB genomes and therefore was likely present in the SB ancestor prior to establishment of the symbiosis. *cypA* is not possessed by any close relatives, is generally rare in *Cyanobacteria*, and is more closely related to eukaryotic than other bacterial *cypA* sequences [48]. Although its function in SBs is not known, *cypA* of intracellular bacterial pathogens can influence host signal transduction and may be involved in iron regulation [48]. Additionally, all SBs have retained all genes necessary for nitrogen fixation, including iron-molybdenum cofactor (FeMo-co) synthesis and molybdate uptake and iron (II) transporters. SBs have also lost genes for acquiring and using other forms of N, including those for assimilatory nitrate uptake and reduction, and the genes for molybdenum cofactor, which is required for nitrate reductase. Consequently, FeMo-co represents the only Mo requirement in SBs. Additionally, SBs have lost nearly all other transporter genes. We summarize other notable gene presence and absence in Supplementary Fig. 2.

The most substantial difference among SB genomes is the pathway for vitamin B_12_ synthesis, which is complete in RulaSB and RgibSB but almost entirely missing in EturSB and EadnSB (Fig. 2B). It is possible that SBs with an intact pathway provide B_12_ to their hosts along with N. *Cyanobacteria* make a different form of B_12_ (pseudocobalamin) than that typically used by eukaryotic algae (cobalamin); at least some diatoms can use pseudocobalamin, but it is less bioavailable than cobalamin [49]. The pathway for B_12_ synthesis is long (17 genes), so the complete maintenance of the pathway in only some SBs is intriguing. All SB genomes still have the B_12_-dependent methionine synthase *metH* and lack the B_12_-independent alternative. This means that SBs without a complete B_12_ pathway must either get B_12_ or methionine from the host.

It is likely that the remaining B_12_ genes will be lost in EturSB and EadnSB. The loss of *cobU* in EturSB but not EadnSB is consistent with ongoing stochastic loss (Fig. 2B). A second possibility is that the host provides SBs with intermediate products. We consider this is an unlikely, as diatoms are not known to produce pseudocobalamin or cobalamin [49] and would require successful transfer of these genes into the host nuclear genome and integration into expression networks. These possibilities could be clarified further with comparative analysis of more SB genomes, host nuclear genome sequencing, and the determination of B_12_ requirements of SB-containing diatoms. This distinct difference in SB genome content across recently diverged host lineages is worth exploring further and may explain differences in host species distributions.

### Transcriptional allocation between host organelles and SB

Given that SBs depend on host photosynthate for N-fixation, we hypothesized that relative chloroplast transcriptional effort would be greater in the absence of a preferred N source. To address this, we investigated how transcriptional effort is allocated between host organelles and SBs dependent on photoperiod (light versus dark) and environmental N source (N_2_ versus nitrate). We inoculated eight flasks of N-deplete media with *R. gibba* 17Bon1 for growth on a 12-h light:dark cycle. After 72 h of growing in N-deplete media, we added nitrate to four of the flasks to a final concentration of 1 mM. Twenty-four hours postnitrate addition, we harvested cells for RNA at mid-light period (24 h-L) and then 12 h later at mid-dark period (36 h-D), as many cyanobacterial genes (including *nif* genes in a close free-living relative) exhibit peak expression at either mid-light or mid-dark [50, 51]. We took a second set of light and dark samples beginning 48 h after the first sampling (72 h-L; 84 h-D). A schematic of the experimental design is available in Supplementary Fig. 3.

**Figure 3:**
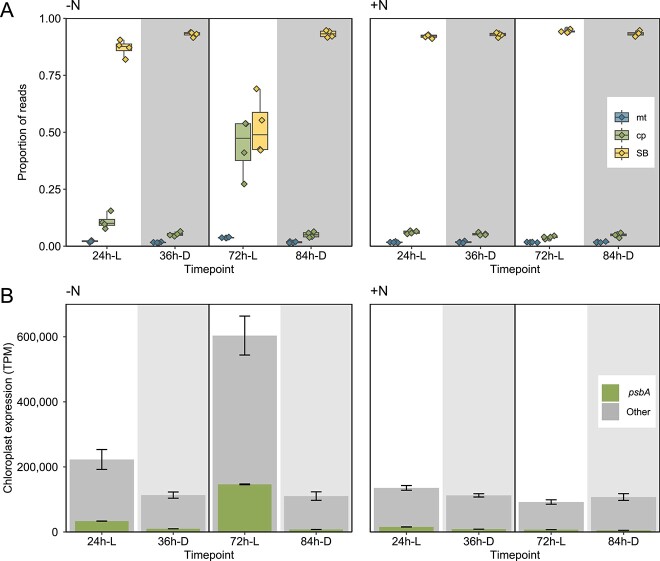
(A) Proportion of reads assigned to chloroplast, mitochondrion, and SB protein-coding genes in −N (left) and + N (right) for all recorded timepoints. mt = mitochondrion; cp = chloroplast (B) chloroplast gene expression in the absence and presence of added nitrate; inset bars represent only *psbA* reads and wider bars represent total transcription for the chloroplast reported as transcript per million (TPM); vertical lines show standard error for both *psbA* transcription and total transcription; for both panels, columns with a white background are light timepoints and a shaded background are dark timepoints.

At all timepoints and in both +N and −N conditions, SB transcripts constitute a significant proportion of reads compared with mitochondrial and chloroplast transcripts (Fig. 3A), suggesting that SBs are a substantial metabolic cost to the host. However, the proportion of SB reads was lower during the day under−N, decreasing the most at 72 h-L. This was due to a massive increase in the relative proportion of chloroplast reads. The increase in chloroplast transcription was largely attributed to increased *psbA* expression (Fig. 3B), a reliable indicator of photosynthetic effort [52]. The addition of nitrate suppressed this effect (Fig. 3A). Our observation of a boost of mid-day N fixation (estimated as acetylene reduction) in the −N treatment is consistent with this interpretation (Supplementary Fig. 4). It is further supported by recent experiments showing that N-fixation rate in SBs is reduced when photosystem (PS) II is suppressed in *Epithemia clementina* [53]. Mitochondrial expression was consistently similar and low throughout. We hypothesize that the upregulation of photosynthesis supports daytime N-fixation in the absence of a preferred N source, demonstrating the high level of metabolic integration between hosts and SBs.

### RgibSB diurnal gene expression is dampened compared with free-living *Cyanobacteria*

How transcription evolves in endosymbionts as they become integrated with their hosts is not well understood. Nutritional endosymbionts of cicadas have an overall weakened ability to regulate gene expression [54], but it remains unclear how pervasive this is across host-endosymbiont systems, or how quickly transcriptional control is lost. To address this, we next examined RgibSB transcription in more detail to determine the extent to which it has diverged from free-living *Cyanobacteria* with respect to diurnal regulation of gene expression. Generally, free-living *Cyanobacteria* strongly regulate their metabolism over day–night cycles, with anabolic processes occurring during the day and catabolic processes at night [55, 56]. During the day, cells photosynthesize, fix carbon, and synthesize glycogen to build up energy stores. At night, glycogen is degraded, and cells primarily use the oxidative pentose phosphate pathway (OPPP) instead of glycolysis for sugar catabolism. This generates NADPH as an electron donor both for anabolic pathways, including N-fixation and for protection from reactive oxygen species via glutathione reductase [55]. Single-celled N-fixing *Cyanobacteria* also fix N at night to temporally separate N-fixation from photosynthesis because they are biochemically incompatible processes, as nitrogenase is extremely sensitive to oxygen [56].

Regarding this canonical day–night metabolic regulation, SBs have two key differences compared to free-living relatives. First, because SBs do not have complete PS I or II, they are reliant on their hosts for fixed C, which is produced by the host chloroplast during the day. Second, SBs from *R. gibba* and two marine strains (*Epithemia pelagica* and *Epithemia catenata*) have been shown to fix N during the day, concurrent with host photosynthesis, as well as into the night [15, 16]. Based on these N-fixation experiments in other *Rhopalodiaceae-*SB systems [15, 16] and our own results (Supplementary Fig. 4), we expected that N-fixation genes would be expressed in both light and dark conditions. This altered temporal regulation of N-fixation may be indicative of a broader change in gene regulation of metabolic pathways in SBs, particularly those that are typically regulated over day–night cycles in related free-living *Cyanobacteria*. Alternatively, this pattern could be limited to only N-fixing pathways. Additionally, by adding nitrate to some samples, we were also able to measure how SBs respond to a pulse of preferred N from the environment.

We tested the differences in expression between both light timepoints and both dark timepoints separately for +N and −N (Supplementary Table 3, models t01–t04). In +N, we found no major differences between 24 h-L versus 72 h-L or 36 h-D versus 84 h-D. However, in −N, there were many significant differences in gene expression between the 24 h-L and 72 h-L (Supplementary Table 3), possibly due to continuing growth in N-deplete media. To constrain our results to differences in N and light conditions, we focused further analyses and discussion on expression data from 72 h-L and 84 h-D.

**Figure 4:**
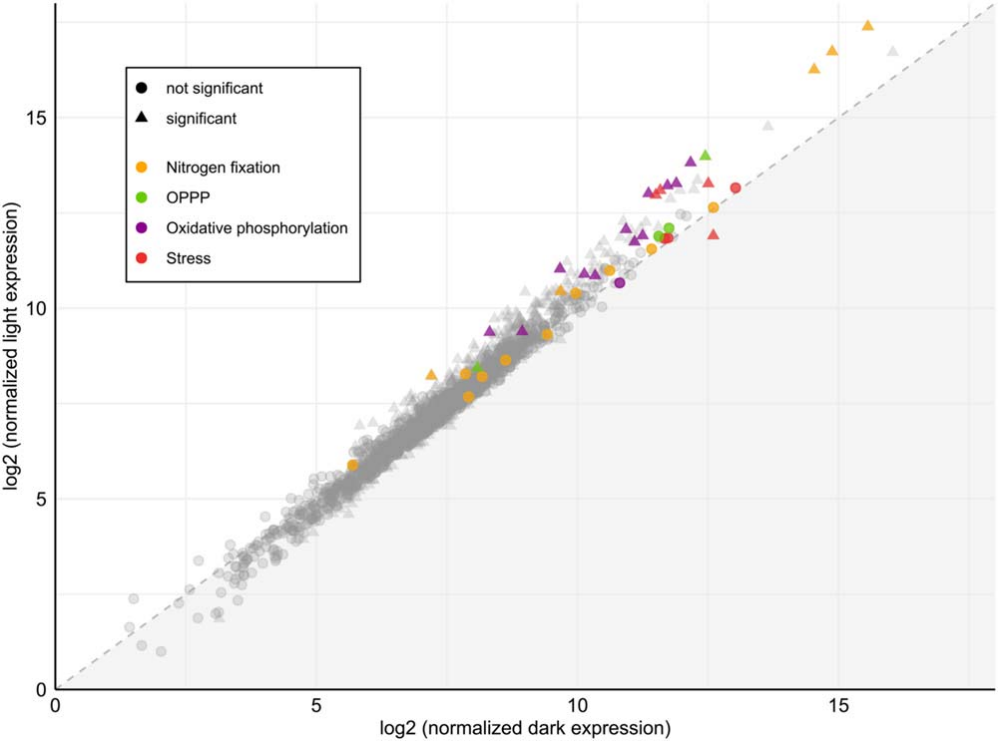
Gene expression of RgibSB 17Bon1 as log2 normalized expression in −N at noon day 3 (72 h-L) and midnight day 3 (84 h-D).

The relationship between gene expression levels in the light versus the dark was markedly linear for both −N (Fig. 4) and + N (Supplementary Fig. 5) conditions compared to the typical diurnal expression patterns of unicellular *Cyanobacteria* at mid-dark and mid-light. In −N, 216 genes were expressed significantly higher in the light, and 55 genes in the dark (adjusted *P* value <.05; Supplementary Table 3, model t08). In +N, 265 genes were expressed significantly higher in the light and 123 genes in the dark (adjusted *P* value <.05; Supplementary Table 3, model t07). No gene had an expression fold change greater than 3.61 or 6.28 for −N and + N, respectively. These differences are very small in magnitude compared with the typical diurnal expression patterns of free-living *Cyanobacteria*, which often have maximum fold changes well above 100 between mid-dark and mid-light periods [50, 51]. When combining both +N and −N data in our model (~ N condition + light), 371 genes had significantly higher expression during the light and 268 genes in the dark (adjusted *P* value <.05; Supplementary Table 3, model t09). Only 43 of these genes were expressed at least 2-fold higher during mid-light, and only three genes had 2-fold higher expression in mid-dark.

We obtained evidence for an interaction between light environment and N availability for a small number of genes (N = 11 with adjusted *P* value <.05; Supplementary Table 3, model t11). Seven of the top 25 genes with a detectable interaction are involved in the maturation of the nitrogenase enzyme (Supplementary Fig. 6). For all seven of these genes, light and dark expression were the same in the absence of nitrate; in +N, however, dark expression was lower. This pattern suggests a shift in nitrogenase regulation in response to nitrate addition that is potentially related to the differences in N-fixation activity that we observed between −N and + N cells (Supplementary Fig. 4). Together, these data suggest that SBs are less responsive to light–dark cycles or have a much weaker ability to regulate gene expression over day–night cycles than their free-living counterparts. Whether this dampening is due to a lack of environmental cues, or a disruption of circadian regulation remains to be determined.

We identified the most highly expressed genes in RgibSB and examined the expression patterns of those typically expected to cycle diurnally. Molecular chaperones including *groES*, *groEL*, and *dnaK* were constitutively among the most highly expressed genes. This pattern is expected in endosymbionts, because their genomes are subject to relatively strong genetic drift and the fixation of slightly deleterious nonsynonymous mutations; consequently, proteins encoded by endosymbionts are often highly susceptible to misfolding [18]. N-fixation genes were also highly expressed compared to most other genes in both light and dark conditions, as expected given that SBs fix N during both day and night (Supplementary Fig. 4; [15, 16]). However, most *nif* genes were still expressed significantly more highly at mid-light than at mid-dark, with the core nitrogenase genes *nifHDK* exhibiting some of the highest fold-changes in gene expression (3–4x) in both +N and −N. However, these changes are modest compared with what has been reported for closely related free-living *Cyanobacteria* (from 20 to >100 fold [50, 51]; Supplementary Fig. 7).

The most highly expressed gene in all time points and all conditions is annotated as a hypothetical protein (Supplementary  Table 4). This gene is present in all four SB genomes and is homologous to a circadian-oscillating polypeptide 23 (COP23) domain-containing protein in *R. orientalis* PCC8801, but its function is unknown. COP23 was first described for its “beautiful circadian oscillating pattern” in transcript, synthesis, and protein abundance [57]. The fact that the COP23 homolog in RgibSB has no detectable difference in expression further supports that SBs have overall weakened diurnal control of gene expression. For many other genes canonically expressed more highly at mid-dark in free-living relatives, these genes were instead more highly expressed at mid-light timepoints in Rgib 17Bon1 SB (Fig. 4). This includes genes involved in both OPPP (2 of 4 genes) and oxidative phosphorylation (12 of 13 genes). Increased daytime expression of canonically dark expressed genes and increased daytime N-fixation suggest that SB N-fixation is tied directly to host photosynthesis during the day and relies on host-stored carbohydrates to power N-fixation at night. The cyanobacterium UCYN-A, a related but independently evolved, single-celled, N-fixing obligate endosymbiont of some marine haptophytes (Fig. 1C), has also shifted to daytime N-fixation [58]. UCYN-A also couples catabolism to the energy produced by photosynthesis in its host [58]. This study also proposed that it may be beneficial to directly link N-fixation and photosynthesis in the oligotrophic ocean, as this is also seen in non-heterocystous, free-living cyanobacterium *Trichodesmium* species [58]. It is possible that a similar mechanism has convergently evolved in SBs and their hosts.

## Conclusion

Our results show that SB metabolism has been significantly altered since transitioning to an endosymbiotic lifestyle. First, SBs appear to have weaker diurnal control over gene expression compared with free-living relatives. Second, observed cases of differential expression indicate a shift to greater daytime catabolism concurrent with N-fixation, likely to support the SB’s central role of providing its host with usable N. This study highlights that endosymbionts can become remarkably integrated with their hosts at the metabolic level over short evolutionary periods as illustrated by their coordination with host photosynthesis. The convergent evolution of daytime N-fixation in SBs and UCYN-A, linking the process to host photosynthesis, suggests that this may be a relatively simple way for hosts to regulate N-fixing endosymbiont metabolism early in the relationship by controlling the cellular energy status of the endosymbiont.

## Supplementary Material

Supplemental_Figures_and_Tables_wrae064

## Data Availability

The datasets generated during and/or analysed during the current study are deposited at NCBI under BioProject PRJNA690824. Raw reads for *R. gibba* 17Bon1 genome assembly are available in the NCBI SRA repository (SRA accession numbers: SRR26035200 through SRR26035202). Raw reads for *E. adnata* 19Bon2 genome assembly are available in the NCBI SRA repository under SRA accession number SRR25945676. RNAseq reads are available in the NCBI SRA repository under accession number SRP459152 (SRA accession numbers SRR26379907 through SRR26379932).

## References

[1] Mann DG . The species concept in diatoms. Phycologi*a* 1999;38:437–95. 10.2216/i0031-8884-38-6-437.1

[2] Villareal TA , Altabet MA, Culver-Rymsza K. Nitrogen transport by vertically migrating diatom mats in the North Pacific Ocean. Natur*e* 1993;363:709–12. 10.1038/363709a0

[3] Foster RA , Zehr JP. Diversity, genomics, and distribution of phytoplankton-cyanobacterium single-cell symbiotic associations. Ann Rev Microbio*l* 2019;73:435–56. 10.1146/annurev-micro-090817-062650 31500535

[4] Foster RA , Kuypers MMM, Vagner T et al. Nitrogen fixation and transfer in open ocean diatom–cyanobacterial symbioses. ISME *J* 2011;5:1484–93. 10.1038/ismej.2011.26 21451586 PMC3160684

[5] Harke MJ , Frischkorn KR, Haley ST et al. Periodic and coordinated gene expression between a diazotroph and its diatom host. ISME *J* 2019;13:118–31. 10.1038/s41396-018-0262-2 30116042 PMC6299110

[6] Karl DM , Church MJ, Dore JE et al. Predictable and efficient carbon sequestration in the North Pacific Ocean supported by symbiotic nitrogen fixation. Proc Natl Acad Sci U S *A* 2012;109:1842–9. 10.1073/pnas.1120312109 22308450 PMC3277559

[7] Subramaniam A , Yager PL, Carpenter EJ et al. Amazon River enhances diazotrophy and carbon sequestration in the tropical North Atlantic Ocean. Proc Natl Acad Sci U S *A* 2008;105:10460–5. 10.1073/pnas.0710279105 18647838 PMC2480616

[8] Inomura K , Follett CL, Masuda T et al. Carbon transfer from the host diatom enables fast growth and high rate of N2 fixation by symbiotic heterocystous *Cyanobacteria*. Plan Theor*y* 2020;9:192. 10.3390/plants9020192 PMC7076409 32033207

[9] Follett CL , Dutkiewicz S, Karl DM et al. Seasonal resource conditions favor a summertime increase in North Pacific diatom–diazotroph associations. ISME *J* 2018;12:1543–57. 10.1038/s41396-017-0012-x 29449611 PMC5955908

[10] Hilton JA , Foster RA, James Tripp H et al. Genomic deletions disrupt nitrogen metabolism pathways of a cyanobacterial diatom symbiont. Nat Commu*n* 2013;4:1767. 10.1038/ncomms2748 23612308 PMC3667715

[11] Benson ME , Kociolek JP, Spaulding SA et al. Pre-Neogene non-marine diatom biochronology with new data from the late Eocene Florissant Formation of Colorado, USA. Stratigraph*y* 2012;9:131–52. 10.29041/strat.09.2.02

[12] Bahls LL , Weber EE. Ecology and distribution in Montana of *Epithemia sorex* Kutzing, a common nitrogen-fixing diatom. Proc Mont Acad Sc*i U S A* 1988;48:15–20

[13] Sculley JB , Lowe RL, Nittrouer CA et al. Eighty years of food-web response to interannual variation in discharge recorded in river diatom frustules from an ocean sediment core. Proc Natl Acad Sci U S *A* 2017;114:10155–9. 10.1073/pnas.1611884114 28874576 PMC5617238

[14] Furey PC , Lowe RL, Power ME et al. Midges, *Cladophora*, and epiphytes: shifting interactions through succession. Freshw Sc*i* 2012;31:93–107. 10.1899/11-021.1

[15] Schvarcz CR , Wilson ST, Caffin M et al. Overlooked and widespread pennate diatom-diazotroph symbioses in the sea. Nat Commu*n* 2022;13:799. 10.1038/s41467-022-28065-6 35145076 PMC8831587

[16] Prechtl J , Kneip C, Lockhart P et al. Intracellular spheroid bodies of *Rhopalodia gibba* have nitrogen-fixing apparatus of cyanobacterial origin. Mol Biol Evo*l* 2004;21:1477–81. 10.1093/molbev/msh086 14963089

[17] Nakayama T , Ikegami Y, Nakayama T et al. Spheroid bodies in rhopalodiacean diatoms were derived from a single endosymbiotic cyanobacterium. J Plant Re*s* 2011;124:93–7. 10.1007/s10265-010-0355-0 20512519

[18] McCutcheon JP , Moran NA. Extreme genome reduction in symbiotic bacteria. Nat Rev Microbio*l* 2012;10:13–26. 10.1038/nrmicro2670 22064560

[19] Kamakura S , Mann DG, Nakamura N et al. Inheritance of spheroid body and plastid in the raphid diatom *Epithemia* (*Bacillariophyta*) during sexual reproduction. Phycologi*a* 2021;60:265–73. 10.1080/00318884.2021.1909399

[20] Nakayama T , Kamikawa R, Tanifuji G et al. Complete genome of a nonphotosynthetic cyanobacterium in a diatom reveals recent adaptations to an intracellular lifestyle. Proc Natl Acad Sci U S *A* 2014;111:11407–12. 10.1073/pnas.1405222111 25049384 PMC4128115

[21] Husnik F , Tashyreva D, Boscaro V et al. Bacterial and archaeal symbioses with protists. Curr Bio*l* 2021;31:R862–77. 10.1016/j.cub.2021.05.049 34256922

[22] Moran NA , McCutcheon JP, Nakabachi A. Genomics and evolution of heritable bacterial symbionts. Annu Rev Gene*t* 2008;42:165–90. 10.1146/annurev.genet.41.110306.130119 18983256

[23] Miller SR , Abresch HE, Ulrich NJ et al. Bacterial adaptation by a transposition burst of an invading IS element. Mol Biol Evo*l* 2021;13:evab245. 10.1093/gbe/evab245 PMC8763236 34791212

[24] Bankevich A , Nurk S, Antipov D et al. SPAdes: a new genome assembly algorithm and its applications to single-cell sequencing. J Comput Bio*l* 2012;19:455–77. 10.1089/cmb.2012.0021 22506599 PMC3342519

[25] Langmead B , Salzberg SL. Fast gapped-read alignment with bowtie 2. Nat Method*s* 2012;9:357–9. 10.1038/nmeth.1923 22388286 PMC3322381

[26] Tatusova T , DiCuccio M, Badretdin A et al. NCBI prokaryotic genome annotation pipeline. Nucleic Acids Re*s* 2016;44:6614–24. 10.1093/nar/gkw569 27342282 PMC5001611

[27] Emms DM , Kelly S. OrthoFinder: phylogenetic orthology inference for comparative genomics. Genome Bio*l* 2019;20:238. 10.1186/s13059-019-1832-y 31727128 PMC6857279

[28] Hoang DT , Chernomor O, von Haeseler A et al. UFBoot2: improving the ultrafast bootstrap approximation. Mol Biol Evo*l* 2018;35:518–22. 10.1093/molbev/msx281 29077904 PMC5850222

[29] Nguyen L-T , Schmidt HA, von Haeseler A et al. IQ-TREE: a fast and effective stochastic algorithm for estimating maximum-likelihood phylogenies. Mol Biol Evo*l* 2015;32:268–74. 10.1093/molbev/msu300 25371430 PMC4271533

[30] Kalyaanamoorthy S , Minh BQ, Wong TKF et al. ModelFinder: fast model selection for accurate phylogenetic estimates. Nat Method*s* 2017;14:587–9. 10.1038/nmeth.4285 28481363 PMC5453245

[31] Letunic I , Bork P. Interactive Tree of life (iTOL) v4: recent updates and new developments. Nucleic Acids Re*s* 2019;47:W256–9. 10.1093/nar/gkz239 30931475 PMC6602468

[32] Darling AE , Mau B, Perna NT. progressiveMauve: multiple genome alignment with gene gain, loss and rearrangement. PLoS On*e* 2010;5:e11147. 10.1371/journal.pone.0011147 20593022 PMC2892488

[33] Page AJ , Cummins CA, Hunt M et al. Roary: rapid large-scale prokaryote pan genome analysis. Bioinformatic*s* 2015;31:3691–3. 10.1093/bioinformatics/btv421 26198102 PMC4817141

[34] Huerta-Cepas J , Szklarczyk D, Heller D et al. eggNOG 5.0: a hierarchical, functionally and phylogenetically annotated orthology resource based on 5090 organisms and 2502 viruses. Nucleic Acids Re*s* 2019;47:D309–14. 10.1093/nar/gky1085 30418610 PMC6324079

[35] Kanehisa M , Sato Y. KEGG mapper for inferring cellular functions from protein sequences. Protein Sc*i* 2020;29:28–35. 10.1002/pro.3711 31423653 PMC6933857

[36] Bolger AM , Lohse M, Usadel B. Trimmomatic: a flexible trimmer for Illumina sequence data. Bioinformatic*s* 2014;30:2114–20. 10.1093/bioinformatics/btu170 24695404 PMC4103590

[37] Magoč T , Salzberg SL. FLASH: fast length adjustment of short reads to improve genome assemblies. Bioinformatic*s* 2011;27:2957–63. 10.1093/bioinformatics/btr507 21903629 PMC3198573

[38] Kopylova E , Noé L, Touzet H. SortMeRNA: fast and accurate filtering of ribosomal RNAs in metatranscriptomic data. Bioinformatic*s* 2012;28:3211–7. 10.1093/bioinformatics/bts611 23071270

[39] Anders S , Pyl PT, Huber W. HTSeq—a python framework to work with high-throughput sequencing data. Bioinformatic*s* 2015;31:166–9. 10.1093/bioinformatics/btu638 25260700 PMC4287950

[40] Love MI , Huber W, Anders S. Moderated estimation of fold change and dispersion for RNA-seq data with DESeq2. Genome Bio*l* 2014;15:550. 10.1186/s13059-014-0550-8 25516281 PMC4302049

[41] Nakayama T , Inagaki Y. Genomic divergence within non-photosynthetic cyanobacterial endosymbionts in rhopalodiacean diatoms. Sci Re*p* 2017;7:13075. 10.1038/s41598-017-13578-8 29026213 PMC5638926

[42] Mareš J , Johansen JR, Hauer T et al. Taxonomic resolution of the genus *Cyanothece* (*Chroococcales*, *Cyanobacteria*), with a treatment on *Gloeothece* and three new genera, *Crocosphaera*, *Rippkaea*, and *Zehria*. J Phyco*l* 2019;55:578–610. 10.1111/jpy.12853 30830691

[43] Bothe H , Tripp HJ, Zehr JP. Unicellular *Cyanobacteria* with a new mode of life: the lack of photosynthetic oxygen evolution allows nitrogen fixation to proceed. Arch Microbio*l* 2010;192:783–90. 10.1007/s00203-010-0621-5 20803290

[44] Bandyopadhyay A , Elvitigala T, Welsh E et al. Novel metabolic attributes of the genus *Cyanothece*, comprising a group of unicellular nitrogen-fixing *Cyanobacteria*. mBi*o* 2011;2:mbio.00214-11. 10.1128/mBio.00214-11 PMC3187577 21972240

[45] Mira A , Ochman H, Moran NA. Deletional bias and the evolution of bacterial genomes. Trends Gene*t* 2001;17:589–96. 10.1016/S0168-9525(01)02447-7 11585665

[46] Campbell MA , Łukasik P, Simon C et al. Idiosyncratic genome degradation in a bacterial endosymbiont of periodical cicadas. Curr Bio*l* 2017;27:3568–3575.e3. 10.1016/j.cub.2017.10.008 29129532 PMC8879801

[47] Perreau J , Moran NA. Genetic innovations in animal–microbe symbioses. Nat Rev Gene*t* 2022;23:23–39. 10.1038/s41576-021-00395-z 34389828 PMC8832400

[48] Wang P , Heitman J. The cyclophilins. Genome Bio*l* 2005;6:226. 10.1186/gb-2005-6-7-226 15998457 PMC1175980

[49] Helliwell KE , Lawrence AD, Holzer A et al. *Cyanobacteria* and eukaryotic algae use different chemical variants of vitamin B12. Curr Bio*l* 2016;26:999–1008. 10.1016/j.cub.2016.02.041 27040778 PMC4850488

[50] Shi T , Ilikchyan I, Rabouille S et al. Genome-wide analysis of diel gene expression in the unicellular N2-fixing cyanobacterium *Crocosphaera watsonii* WH 8501. ISME *J* 2010;4:621–32. 10.1038/ismej.2009.148 20107492

[51] Stockel J , Welsh EA, Liberton M et al. Global transcriptomic analysis of *Cyanothece* 51142 reveals robust diurnal oscillation of central metabolic processes. Proc Natl Acad Sci U S *A* 2008;105:6156–61. 10.1073/pnas.0711068105. 18427117 PMC2329701

[52] Pfannschmidt T , Nilsson A, Allen JF. Photosynthetic control of chloroplast gene expression. Natur*e* 1999;397:625–8. 10.1038/17624

[53] Moulin SLY , Frail S, Braukmann T et al. The endosymbiont of Epithemia clementina is specialized for nitrogen fixation within a photosynthetic eukaryote. ISME Commun 2024; ycae055. 10.1093/ismeco/ycae055 PMC11070190 38707843

[54] Spencer N , Łukasik P, Meyer M et al. No transcriptional compensation for extreme gene dosage imbalance in fragmented bacterial endosymbionts of cicadas. Genome Biol Evo*l* 2023;15:evad100. 10.1093/gbe/evad100 37267326 PMC10287537

[55] Welkie DG , Rubin BE, Diamond S et al. A hard day’s night: *Cyanobacteria* in diel cycles. Trends Microbio*l* 2019;27:231–42. 10.1016/j.tim.2018.11.002 30527541 PMC6377297

[56] Sherman LA , Meunier P, Colon-Lopez MS. Diurnal rhythms in metabolism: a day in the life of a unicellular, diazotrophic cyanobacterium. Photosynth Re*s* 1998;58:25–42. 10.1023/A:1006137605802

[57] Chen H-M , Chien C-Y, Huang T-C. Regulation and molecular structure of a circadian oscillating protein located in the cell membrane of the prokaryote *Synechococcus* RF-1. Plant*a* 1996;199:520–7 8818292 10.1007/BF00195182

[58] del C Muñoz-Marín M , Shilova IN, Shi T et al. The transcriptional cycle is suited to daytime N_2_ fixation in the unicellular cyanobacterium “Candidatus Atelocyanobacterium thalassa” (UCYN-A). mBi*o* 2019;10:e02495–18. 10.1128/mBio.02495-18 30602582 PMC6315102

[59] Shih PM , Wu D, Latifi A et al. Improving the coverage of the cyanobacterial phylum using diversity-driven genome sequencing. Proc Natl Acad Sci U S *A* 2013;110:1053–8. 10.1073/pnas.1217107110 23277585 PMC3549136

