# Supplementary material for: Diurnal transcriptional variation is reduced in a nitrogen-fixing diatom endosymbiont
Source: ISME J. 2024 Apr 18;18(1):wrae064. doi: 10.1093/ismejo/wrae064 (PMC11131595; doi:10.1093/ismejo/wrae064)
Supplement: Supplemental_Figures_and_Tables_wrae064 [file supplemental_figures_and_tables_wrae064.pdf]

## Supplemental Figures

**Supplemental Figure 1:** The chromosomes of (A) R. gibba 17Bon1 SB and **(B)** *E. adnata* 19Bon2 SB. Scale is in megabases. Grey bars represent predicted genes, orange represents predicted pseudogenes, and green represents ribsomal operons.

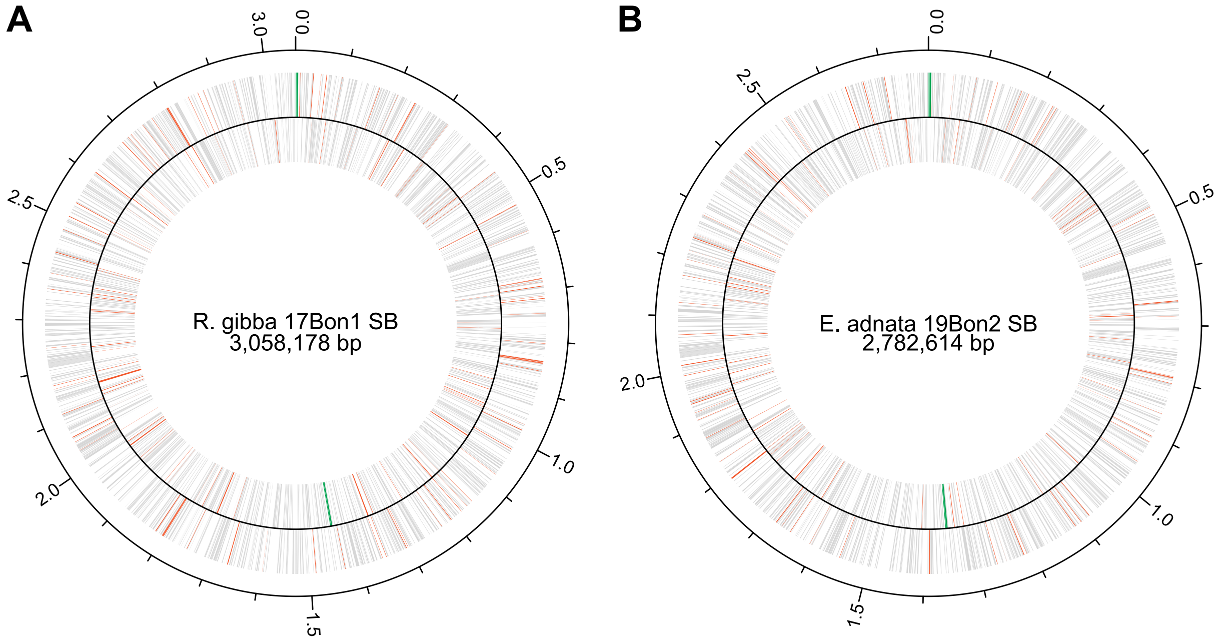

**Supplemental Figure 2:** Noted presence and absence of pathways in SBs. SB expanded gene presence and absence plot including amino acids, DNA repair and recombination, also core carbon pathways if not in main text, core carbon pathways, OPPP and RPPP, nitrogen metabolism, ion and nutrient uptake, MoCo Fe-MoCo and membrane transport.

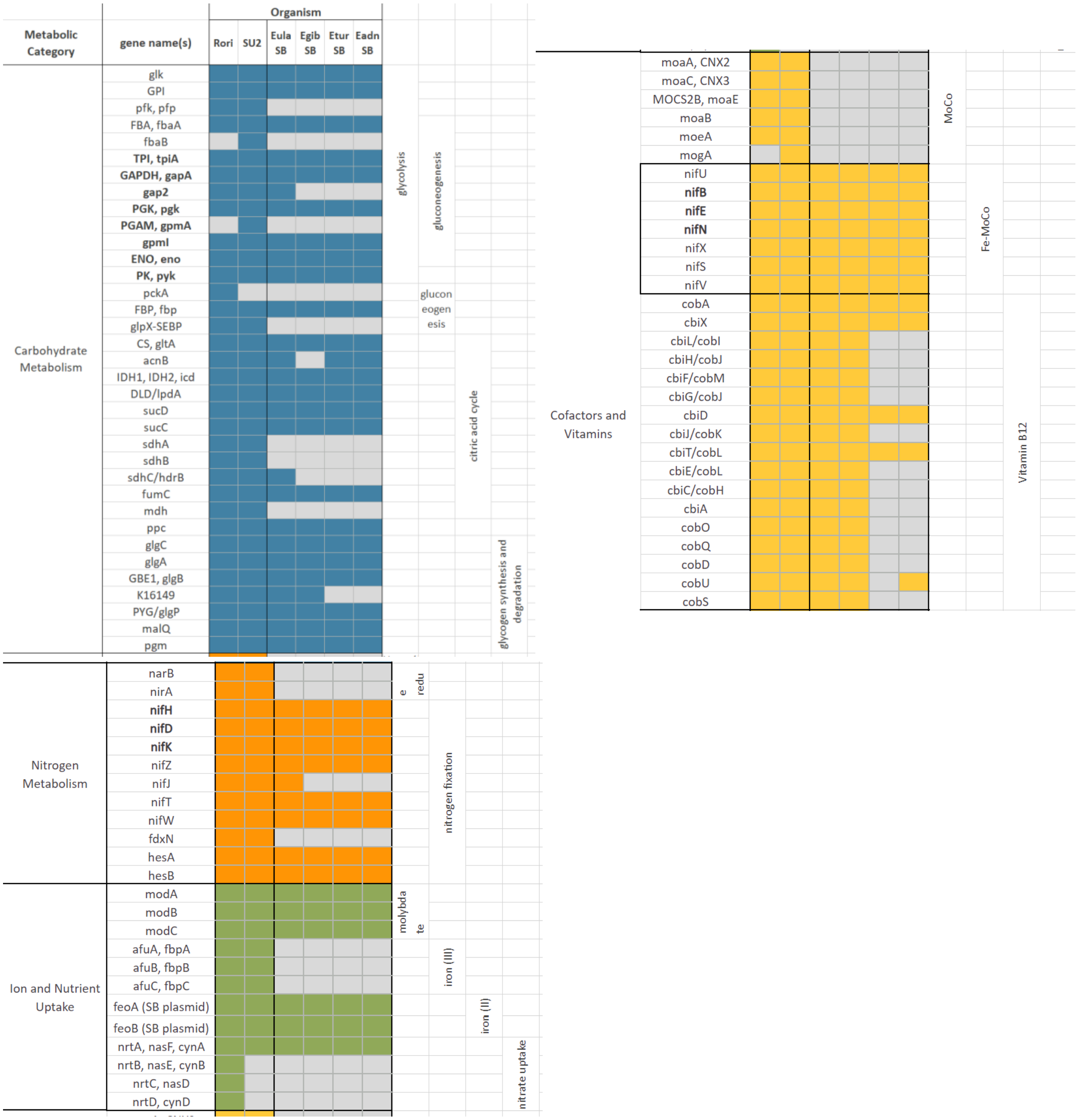

**Supplemental Figure 3: A)** Schematic of experimental design for transcriptome sampling for *R. gibba* 17Bon1. **B)** Cartoon of *R. gibba* system and proposed interactions between host photosynthesis and SBs.

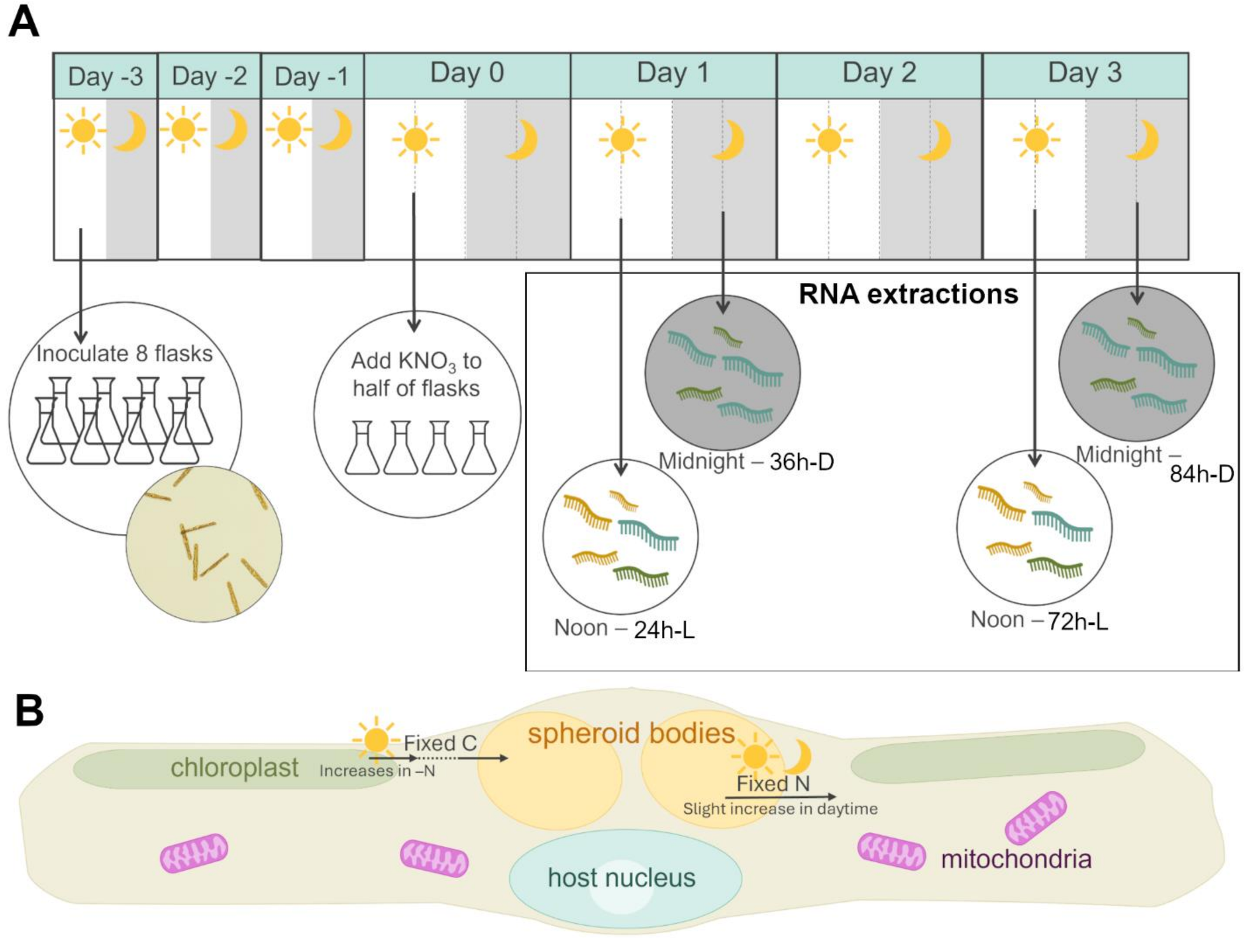

**Supplemental Figure 4:** Nitrogen fixation of Rgib 17Bon1 SB as measured via acetylene reduction. There is a significant interaction between N treatment and light availability ($t$ = 2.87, $P$ < 0.01). Bars are mean values for quadruplicate samples across both light (24h-L and 72h-6) and dark (36h-D and 84h-D) time points, and error bars represent standard error. The assay was conducted by adding 5 mL of cells at equal cell concentrations ($OD_{750}$ = 0.04) to 20 mL crimp seal vials four hours before each RNA-sampling timepoint. Vials were crimp sealed, and 5 mL of headspace was removed with a syringe before adding 5 mL of fresh acetylene gas (made by mixing calcium carbide and water). After four hours of incubation, the headspace was removed and added to empty 5 mL crimp sealed vials and stored upside-down in water until measuring ethylene concentrations by gas chromatography the following day (less than 36 hours after sampling).

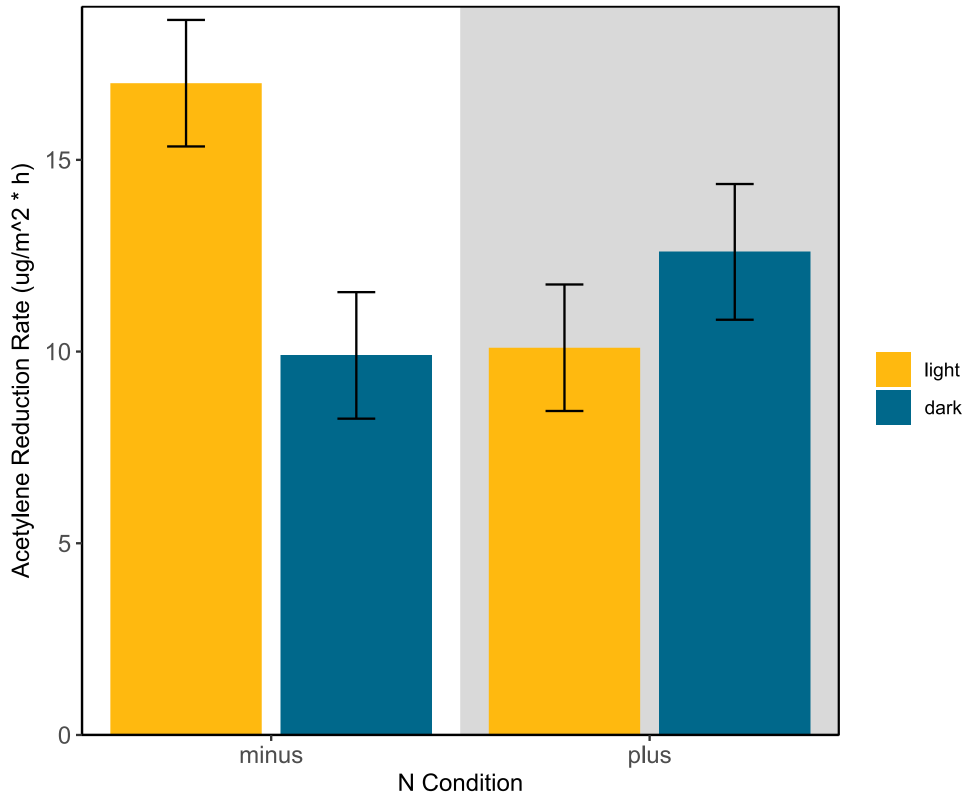

**Supplemental Figure 5:** Gene expression of Rgib 17Bon1 SB as log2 Normalized Expression in +N at 72h-L and 84h-D.

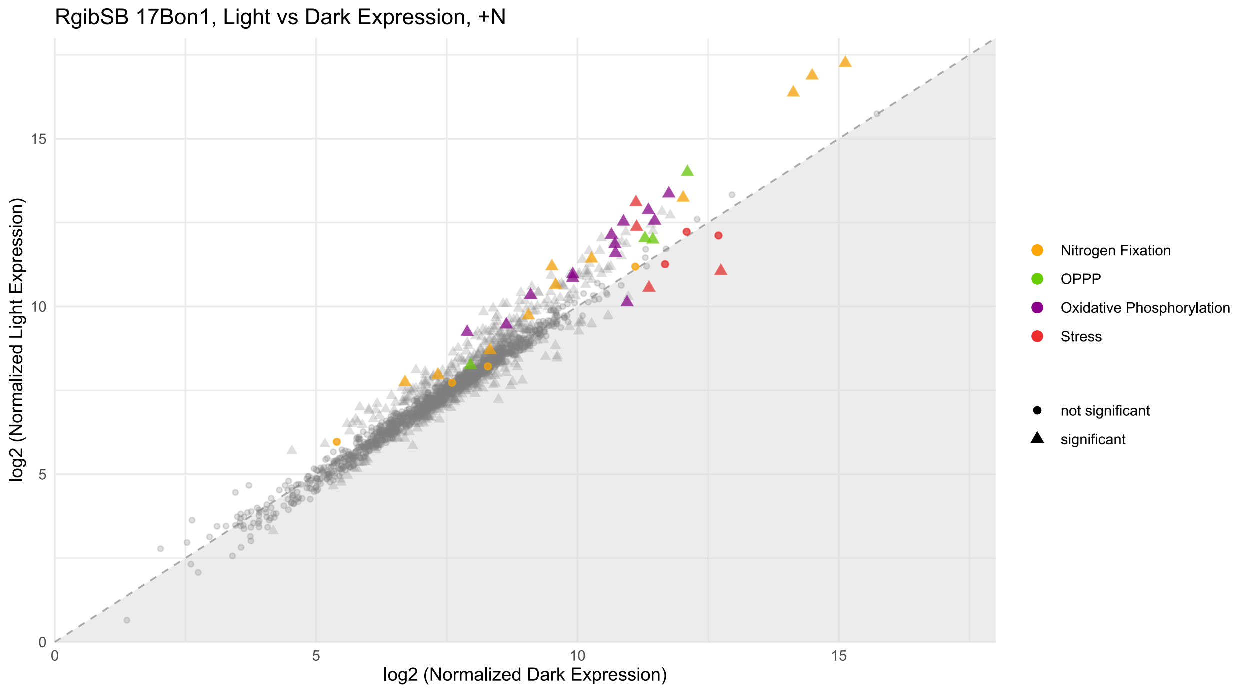

**Supplemental Figure 6:** Light and nitrogen availability interaction plots for the expression of seven genes involved in nitrogenase maturation. Significance values are a result of a likelihood ratio test comparing models of gene expression with an interaction term (~condition + light + condition*light) versus the model without (~condition + light).

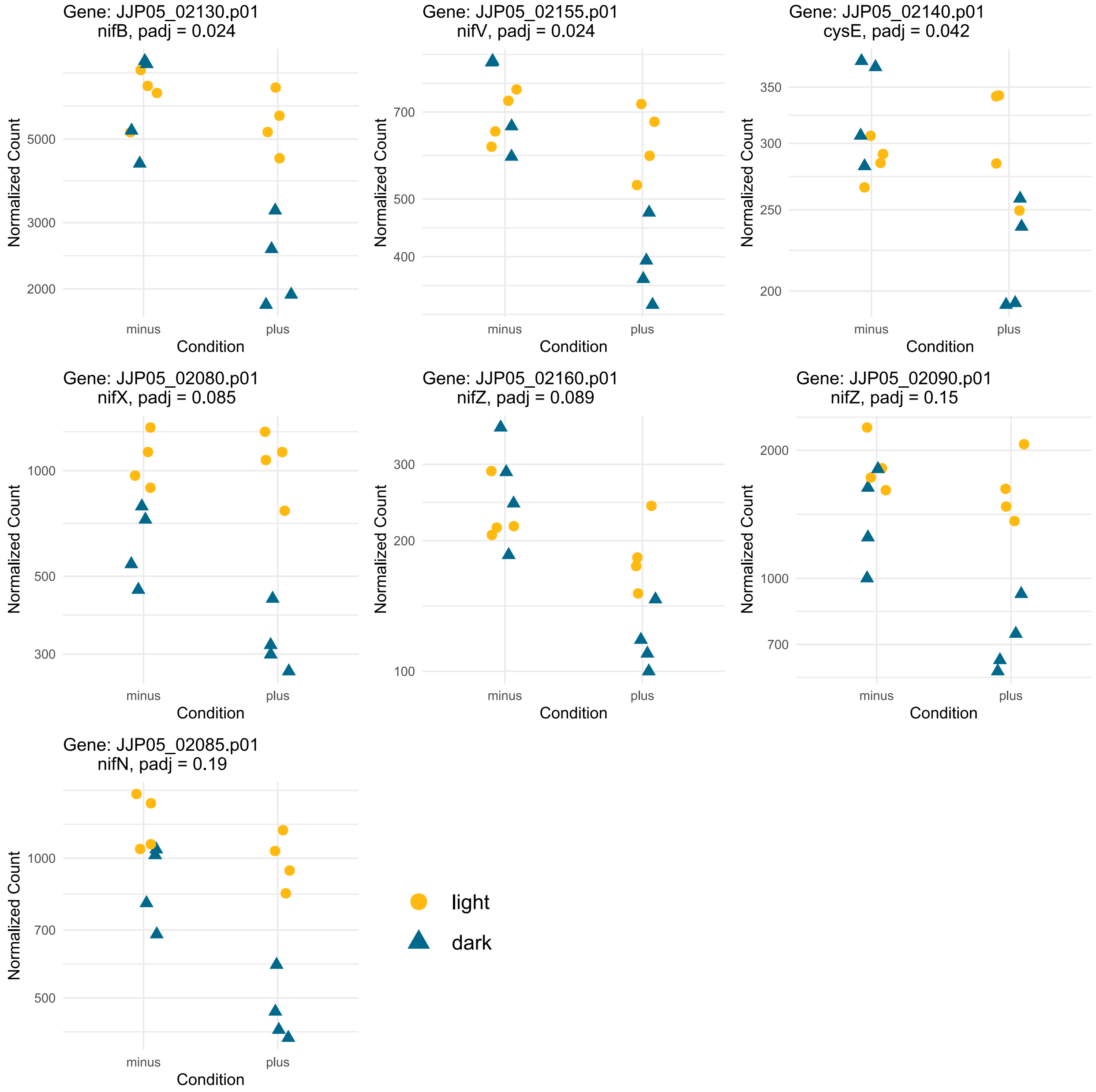

**Supplemental Figure 7**: Total fold change in expression for *nifHDK* between light and dark in RgibSB 17Bon1 compared to free-living relatives. Total fold changes for *C. watsonii* WH8501 and *C. subtropica* ATCC51142 are taken from main text references [28] and [29] respectively.

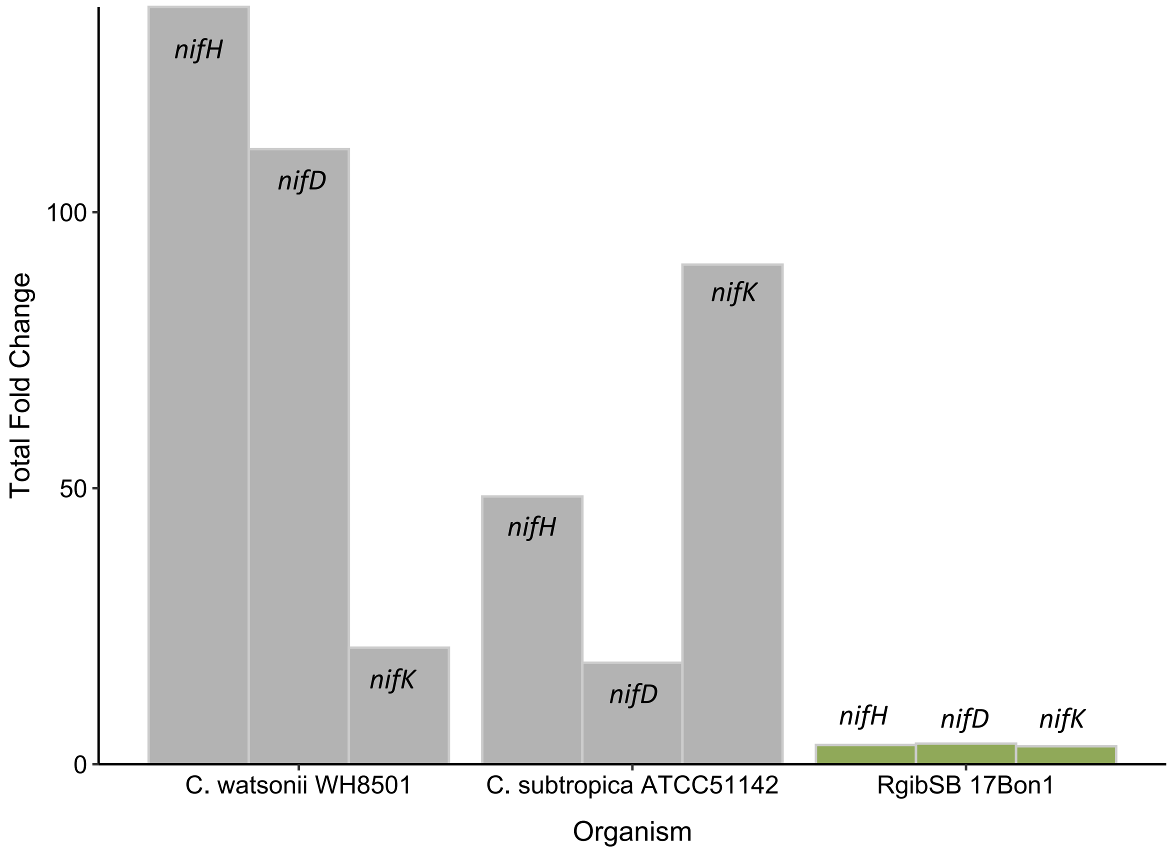

## Supplemental Tables

**Supplemental Table 1:** Mitochondrion and chloroplast genome assembly statistics. Note that for both chloroplast assemblies, the shortest scaffold has twice the coverage of the other two scaffolds and is likely duplicated in the chromosome. The coding genes reported for *R. gibba* 17Bon1 mitochondrial genome annotation was manually curated, while the statistics reported for *E. adnata* 19Bon2 mitochondrial and both chloroplast genomes are from automatic annotations only (see methods in main text).

| ***Genome Information*** | ***E. adnata* 19Bon2** | ***R. gibba* 17Bon1** |
|---|---|---|
| | ***Mitochondrion*** | |
| **# of scaffolds** | 1 | 1 |
| **Median Coverage** | 854.3x | 34.4x |
| **Length (bp)** | 34,415 | 44,387 |
| **% GC** | 25.2% | 22.9% |
| **Predicted Protein Coding Genes** | 35 | 42 |
| **Accession Number** | OR527426 | OR515804 |
| | ***Chloroplast*** | |
| **# of scaffolds** | 3 | 3 |
| **Median Coverage** | 375.8x | 24.3x |
| **Length (bp)** | 112473 | 112660 |
| **% GC** | 30.3% | 30.30% |
| **Predicted Protein Coding Genes** | 134 | 128 |
| **Accession Number** | OR527427-OR527429 | OR515805-OR515807 |

**Supplemental Table 2:** Details of mobile element fragments remaining in SB genomes. Shared families identified in all SBs are highlighted by the same color.

| Organism | ID | ISFinder Family | ISFinder Top Hit | Length of top hit (aa) | Length of SB sequence (aa) |
|---|---|---|---|---|---|
| EadnSB | KPI85_00185 | IS200/IS605 | ISSs1 | 429 | 157 |
| EadnSB | KPI85_01085 | IS3 | ISAs20 | 405 | 66 |
| EadnSB | KPI85_03805 | IS982 | ISLbp3 | 305 | 36 |
| EadnSB | KPI85_03810 | IS982 | ISPasp1 | 292 | 38 |
| EadnSB | KPI85_04000 | IS982 | ISNeu2 | 208 | 54 |
| EadnSB | KPI85_05720 | IS982 | ISPasp2 | 292 | 51 |
| EadnSB | KPI85_06850 | ISL3 | ISMae36 | 404 | 36 |
| EadnSB | KPI85_08845 | IS3 | IS-LL6 | 385 | 298 |
| RgibSB | JJP05_01175 | ISL3 | ISMae36 | 404 | 30 |
| RgibSB | JJP05_03910 | - | - | - | - |
| RgibSB | JJP05_09470 | IS3 | IS1163 | 87 | 303 |
| EturSB | ETSB_RS04375 | IS3 | IS-LL6 | 385 | 298 |
| EturSB | ETSB_RS09760 | ISL3 | ISAsp1 | 407 | 42 |
| RulaSB | RGRSB_RS04660 | IS3 | IS1163 | 87 | 302 |
| RulaSB | RGRSB_RS05140 | ISL3 | ISMae36 | 404 | 40 |
| RulaSB | RGRSB_RS05915 | - | - | - | - |
| RulaSB | RGRSB_RS08550 | IS630 | ISRm10 | 315 | 70 |
| RulaSB | RGRSB_RS08665 | - | - | - | - |
| RulaSB | RGRSB_RS08880 | ISL3 | ISAsp1 | 407 | 26 |
| RulaSB | RGRSB_RS09560 | IS630 | ISRm10-1 | 311 | 35 |
| RulaSB | RGRSB_RS09665 | - | - | - | - |

**Supplemental Table 3:** Overview of models used in DESeq2 to analyze gene expression in RgibSB 17Bon1. The results for t05 and t06 are not discussed in the main text for brevity but are included here for completeness.

| Test ID | Model | +N/ -N | Light Condition | Timepoints | Number of genes with LFC >0 (higher day/ higher plus N) | Number of genes with LFC <0 (higher night/higher minus N) | Smallest adjusted P-value |
|---|---|---|---|---|---|---|---|
| t01 | ~ day | +N | light | 24h-L, 72h-L | 0 | 0 | 0.989 |
| t02 | ~ day | +N | dark | 36h-D, 84h-D | 0 | 6 | 2.20E-06 |
| t03 | ~ day | -N | light | 24h-L, 72h-L | 237 | 80 | 9.69E-11 |
| t04 | ~ day | -N | dark | 36h-D, 84h-D | 0 | 0 | 0.999 |
| t05 | ~ condition | both | light | 72h-L | 18 | 126 | 1.20E-07 |
| t06 | ~ condition | both | dark | 84h-D | 4 | 107 | 1.67E-10 |
| t07 | ~ light | +N | both | 72h-L, 84h-D | 265 | 123 | 1.35E-30 |
| t08 | ~ light | -N | both | 72h-L, 84h-D | 216 | 55 | 4.01E-18 |
| t09 | ~ condition + light | both | both | 72h-L, 84h-D | 371 | 268 | 3.17E-48 |
| t10 | ~ light + condition | both | both | 72h-L, 84h-D | 90 | 278 | 7.77E-07 |
| t11 | LRT: ~condition + light + condition*light vs. ~condition + light | both | both | 72h-L, 84h-D | 3 | 8 | 0.0145 |

**Supplemental Table 4:** Top 10 most expressed gene for all samples at 72h-L and 84h-D timepoints after read normalization by DESeq2 with the highest expressed gene at the top of the column. Normalized read counts used for -N were generated from model t08 and for +N from model t07. Genes are colored by functional category. When possible, locus IDs are replaced with preferred gene name for clarity as there are no duplicate gene copies with the exception of *groEL*, where *groEL* (1) = JJP05_00415 and *groEL* (2) = JJP05_02790.

| Minus N | | | | | | | | Plus N | | | | | | | |
|---|---|---|---|---|---|---|---|---|---|---|---|---|---|---|---|
| 72h-L | | | | 84h-D | | | | 72h-L | | | | 84h-D | | | |
| Am | Bm | Cm | Dm | Am | Bm | Cm | Dm | Ap | Bp | Cp | Dp | Ap | Bp | Cp | Dp |
| JJP05_06850 | JJP05_06850 | JJP05_06850 | nifH | JJP05_06850 | JJP05_06850 | JJP05_06850 | JJP05_06850 | JJP05_06850 | JJP05_06850 | nifH | nifH | JJP05_06850 | JJP05_06850 | JJP05_06850 | JJP05_06850 |
| nifH | nifH | nifH | JJP05_06850 | nifH | nifH | nifH | nifH | nifH | nifH | JJP05_06850 | JJP05_06850 | nifH | nifH | JJP05_05240 | nifH |
| nifD | nifD | nifD | nifD | nifD | nifD | nifD | nifD | nifD | nifD | nifD | nifD | JJP05_05240 | JJP05_05240 | nifH | JJP05_05240 |
| nifK | nifK | nifK | nifK | nifK | nifK | nifK | nifK | nifK | nifK | nifK | nifK | nifD | groEL (2) | groEL (2) | groEL (2) |
| JJP05_03675 | JJP05_03675 | JJP05_03675 | JJP05_03675 | JJP05_03675 | JJP05_05240 | groEL (2) | groEL (2) | JJP05_03675 | JJP05_03675 | JJP05_03675 | JJP05_03675 | groEL (2) | nifD | JJP05_03675 | nifD |
| groEL (2) | groEL (2) | groEL (2) | gnd | groEL (2) | JJP05_03675 | JJP05_05240 | JJP05_05240 | gnd | gnd | gnd | gnd | nifK | JJP05_03675 | nifD | JJP05_03675 |
| gnd | gnd | gnd | groEL (2) | JJP05_05240 | groEL (2) | JJP05_03675 | JJP05_03675 | JJP05_01540 | JJP05_01540 | nifB | nifB | JJP05_03675 | nifK | nifK | nifK |
| groEL (1) | groEL (1) | nifB | groEL (1) | nifB | nifB | nifB | nifB | groEL (2) | nifB | JJP05_01540 | ctaD | JJP05_01540 | JJP05_01540 | groEL (1) | JJP05_01540 |
| ctaD | ctaD | ctaD | ctaD | groEL (1) | groEL (1) | groEL (1) | JJP05_08655 | nifB | groEL (2) | groEL (2) | groEL (2) | groES | groEL (1) | JJP05_01540 | groEL (1) |
| JJP05_01320 | JJP05_01320 | groEL (1) | JJP05_01320 | JJP05_01540 | JJP05_01540 | JJP05_01540 | groEL (1) | ctaD | ctaD | ctaD | JJP05_01540 | groEL (1) | JJP05_08655 | dnaK | dnaK |

| |
|---|
| hypothetical protein - no predicted function |
| nitrogen fixation |
| stress and molecular chaperones |
| membrane transport |
| oxidative pentose phosphate pathway |
| oxidative phosphorylation |
